# Extracorporeal Life Support for Recurrent Hypothermic Cardiac Arrest: A Case Report

**DOI:** 10.7759/cureus.49684

**Published:** 2023-11-29

**Authors:** Simon Kalisz, Timothée Stoll, Fatima-Zohra Bouazza, Marc Claus, Stefano Malinverni

**Affiliations:** 1 Emergency Department, CHU Saint-Pierre, Brussels, BEL; 2 Intensive Care Unit, CHU Saint-Pierre, Brussels, BEL

**Keywords:** extracorporeal life support, hypothermic cardiac arrest, ecls, cardiac arrest, hypothermia

## Abstract

Hypothermia-associated cardiac arrest (HACA) is a challenge for emergency physicians. Standard cardiopulmonary resuscitation (CPR) remains the primary intervention for the treatment of HACA, but extracorporeal life support (ECLS) may be needed as an adjunct to CPR. In this report, we present the case of an adult Asian patient who experienced two episodes of HACA at a two-year interval. In both episodes, the patient was treated with ECLS in addition to standard CPR. We discuss the fundamentals of HACA and how to safely and effectively incorporate ECLS into its management. No-flow time, age, comorbidities, and the cause of the cardiac arrest are criteria to consider when deciding on the duration of CPR and the intensity of the resources deployed. Hypothermia is a reversible cause of cardiac arrest, justifying prolonged CPR. According to the Hypothermia Outcome Prediction after ECLS (HOPE) score, active rewarming through ECLS is recommended. However, a history of cardiac arrest is rare and might be considered a severe comorbidity contraindicating ECLS use. Nevertheless, the indication is determined on a case-by-case basis.

## Introduction

Hypothermia-associated cardiac arrest (HACA) is a low-incidence, high-risk emergency in adults [1]. Standard cardiopulmonary resuscitation (CPR) remains the cornerstone approach for the treatment of HACA, but extracorporeal life support (ECLS) may be needed infrequently as an adjunct to CPR [2]. Choosing the ideal candidate for ECLS is challenging because of its limited access, complexity, and costs associated with the procedure. The Hypothermia Outcome Prediction after ECLS (HOPE) score, by considering age, sex, presence of asphyxia, CPR duration, serum potassium, and core temperature, provides an accurate estimate of the survival probability in patients with HACA undergoing ECLS rewarming [3]. While comorbidities are usually considered when considering a patient for ECLS [4], the HOPE score fails to incorporate preexisting medical conditions when making recommendations for ECLS treatment in patients with HACA [5]. This potentially exposes the clinician to an ethical dilemma when having to decide on starting ECLS in a patient with a prior potentially severe medical condition [6].

We present the case of a patient who experienced two episodes of HACA, two years apart. The patient was treated with ECLS on both occasions and survived with intact neurological function in both episodes.

## Case presentation

First episode

A 52-year-old homeless patient with a history of alcoholism was admitted to the emergency department (ED) in March 2020 by the emergency medical service (EMS) in the context of drowsiness, clonic movements of the upper limbs, and hypothermia. Witnesses found him drowsy in the street and had some nonprolonged clonic movements in front of the EMS workers. While moving the patient from the prehospital stretcher to the hospital bed, the patient was found to become unresponsive with a lack of pulse and breathing. CPR with manual chest compression and bag-valve-mask ventilation was initiated immediately. The first rhythm assessed was ventricular fibrillation. The first measured rectal temperature with a low reading thermometer was 23.4 °C. Warm fluids and physical warming of the patient were initiated through pulsed hot air covers. Manual chest compressions were rapidly substituted by mechanical chest compression through a LUCAS device (Jolife AB, Lund, Sweden). The first arterial blood gas analysis performed during CPR (10 minutes after the arrest) reported a pH of 7.22, a pCO_2_ of 45 mmHg, and a pO_2_ of 88 mmHg. Potassium levels were 3.8 mmol/L, and lactate levels were 6.8 mmol/L. Airway examination demonstrated reduced mouth opening. After three unsuccessful attempts at intubation, by various emergency doctors, with the same Macintosh blade, intubation was performed with a video-laryngoscope while maintaining CPR. The first end-tidal CO_2_ (EtCO_2_) after tracheal tube placement was 9 mmHg. Treatment with ECLS was considered, and the cardiac surgery department was alerted. In total, the patient stayed in the ED resuscitation room for 72 minutes. During this period, the patient was administered five shocks and received 8 mg of adrenaline, along with 300 mg of amiodarone, per ERC guidelines on special circumstances. ECLS was initiated 120 minutes after the arrest; the patient was rewarmed and adequate flow was generated. After two additional cardioversion attempts, the patient had a return of spontaneous circulation and was transferred to the ICU. The patient was discharged from the ICU after eight days with a complete neurological recovery without any sequelae.

Second episode

In January 2022, the same patient was admitted to the ED via ambulance service for drowsiness and hypothermia, probably induced by Belgium's cold climate in January. The patient was also intoxicated with alcohol. Again, as the patient was moved from the ambulance stretcher to the hospital bed, he suffered from cardiac arrest (CA). CPR maneuvers were immediately initiated with manual chest compressions and bag-mask ventilation. The first rhythm assessed was asystole and changed to ventricular fibrillation after the second two-minute resuscitation cycle. The first measured rectal temperature with a low reading thermometer was 24.6 °C, and the first serum potassium was 3.3 mmol/L. Given the known difficult airway, an I-gel supraglottic airway device (Intersurgical, Wokingham, UK) was inserted. The first recorded EtCO_2_ was 12 mmHg. The patient was heated through pulsed air covers, and three cardioversion attempts were initially made. One milligram of adrenaline and 300 mg of amiodarone were administered, according to the Advanced Cardiovascular Life Support (ACLS) protocol. The decision to start ECLS was taken collectively by the ED physicians, the intensivists, and the cardiac surgeons, after careful consideration of the patient’s history, including alcoholism, homelessness, and previous CA. The extracorporeal flow was achieved 122 minutes after the CA and two additional cardioversion attempts. Warming of the patient was achieved through the extracorporeal circuit. Two additional cardioversion attempts and a dose of 150 mg of amiodarone were necessary to obtain the return of spontaneous circulation. Again, the patient was discharged from the ICU after seven days with minimal physical sequelae and an intact neurological function, without any sequelae.

## Discussion

In this report, we present the case of an adult patient who experienced two episodes of HACA at a two-year interval, which necessitated ECLS in addition to standard CPR. The patient’s outcome was favorable in both cases, and full recovery without any neurological sequelae was achieved. In our case, the patient had many physiologic features contributing to an intact neurological recovery despite two CAs associated with prolonged CPR: (1) the patient was deeply hypothermic before the arrest in both episodes, (2) shockable rhythm was observed throughout most of the two arrests, and (3) the arrests were witnessed in both cases, with virtually zero no-flow interval in both events. On the other hand, the patient had low EtCO_2_ during both episodes, but these were probably associated with the low metabolic activity associated with hypothermia rather than with low cardiac output during chest compressions. We hypothesized that the virtually absent no-flow interval, the presence of a shockable rhythm, a low core temperature before arrest, and no history of asphyxia were essential factors contributing to the successful neurological intact recovery after two separate cardiac arrests. These cases involved prolonged low-flow situations, necessitating two ECLS treatments [7].

Hypothermia

Accidental hypothermia is a reversible cause of CA that is potentially associated with a more favorable neurological outcome compared to patients suffering from CA from other etiologies [8]. The risk of CA starts to increase at temperatures below 32 °C. However, it is not until the central body temperature falls below 28 °C that hypothermia is likely the sole cause of CA [9]. Brain oxygen consumption decreases by approximately 6% per 1 °C in core temperature [10] and drops to 16% at 15 °C compared with normothermia [11].

Management

The published evidence places significant emphasis on the need for careful and gentle movements when handling hypothermic patients to prevent the onset of a life-threatening arrhythmia [9]. In our case, both episodes of CA seem to be associated with swift and sudden movements of the patient from the ambulance stretcher to the hospital bed. Maximal care should, therefore, be exercised whenever mobilizing hypothermic patients.

The European Resuscitation Council (ERC) 2021 guidelines recommend withholding adrenaline administration in accidental HACA and limiting defibrillation to three attempts until the core temperature is >30 °C [12]. By contrast, the American Heart Association guidelines [13] allow further defibrillation attempts concurrent with rewarming strategies and state that it may be reasonable to consider adrenaline administration during CPR according to the standard advanced life support (ALS) algorithm [13] as defibrillation attempts have been successful in patients with a core temperature >24 °C [14].

Finally, HACA is one of the few cases in which mechanical chest compression is recommended or, at least, should be considered due to the prolonged resuscitation efforts and the inevitable fatigue that rescuers might experience and that would decrease the quality of CPR [9]. In both cases, mechanical CPR was used, freeing hands from the nurses and cognitive space from the team leader to prepare for ECLS placement.

ECLS using venous-arterial peripheral extracorporeal membrane oxygenation has been associated with improved outcomes in patients experiencing HACA, with scientific evidence supporting its use as the preferred technique for rewarming during CA [15].

Prognostication

Considering the improved tolerance of the brain to no-flow and low-flow conditions observed during accidental hypothermia, accurate case-specific prognostication rules are recommended to guide these prolonged and resource-intensive resuscitation maneuvers. The HOPE score focuses on patients with CA who underwent ECLS rewarming. Age, sex, presence of asphyxia, CPR duration, serum potassium, and core temperature are aggregated to estimate the chances of survival after rewarming and ECLS support [3]. The negative predictive value of a HOPE probability <10% was 97%, which suggests sufficient discrimination and guidance on not initiating ECLS. The HOPE score might, therefore, guide clinicians in the decision on whether to start ECLS rewarming in hypothermic arrest, avoiding an underestimation of the patient’s survival probability. For the patient reported in this study, the HOPE score indicated a survival probability of 60% for the first arrest. For the second arrest, the estimated survival probability was 61% when the estimation was done without taking into account a previous arrest. While the HOPE score has not been developed for the eventuality of assessing the probability of a second hypothermic arrest for the same patient, we thought that the best approximation would be to aggregate the no-flow time of the two episodes when estimating the survival probability. By taking into account a no-flow time of 242 minutes, the resulting survival probability, according to the HOPE score, would be 47%, suggesting a potential benefit of initiating an ECLS despite the history of a previous arrest, above the futility threshold that has been set at 10% [16].

The best chance of full recovery from hypothermic CA occurs in the previously well patient with witnessed CA, where continuous CPR is implemented immediately, timely ECLS commences, and appropriate critical care support is available after the return of spontaneous circulation (ROSC).

## Conclusions

The decision to start ECLS in addition to standard CPR remains challenging in patients with HACA. The HOPE score should be used to support the decision on whether to initiate ECLS. While severe medical conditions should be considered when weighing the decision to initiate ECLS, a previous episode of cardiac arrest should not be considered a formal contraindication.

The repeated use of ECLS in HACA without hypoxia appears to be justified to us, with potentially good neurological outcomes, based on limited data from two incidents in this case report. Although the number of similar cases is probably rare, more studies are needed to strengthen these findings.
